# The Sponge-associated Bacterium *Bacillus licheniformis* SAB1: A Source of Antimicrobial Compounds

**DOI:** 10.3390/md8041203

**Published:** 2010-04-09

**Authors:** Prabha Devi, Solimabi Wahidullah, Cheryl Rodrigues, Lisette D. Souza

**Affiliations:** Bioorganic Chemistry Laboratory, Chemical Oceanography Division, National Institute of Oceanography, Council of Scientific and Industrial Research (CSIR), Dona Paula 403004, Goa, India; E-Mails: solima@nio.org (S.W.); cheryl.rodrigues@nio.org (C.R.); lisette@nio.org (L.D.S.)

**Keywords:** sponge, associated bacteria, Bacillus licheniformis, secondary metabolites, antibiotic activity

## Abstract

Several bacterial cultures were isolated from sponge *Halichondria* sp., collected from the Gujarat coast of the Indo Pacific region. These bacterial cultures were fermented in the laboratory (100 mL) and the culture filtrate was assayed for antibiotic activity against 16 strains of clinical pathogens. *Bacillus* sp. (SAB1), the most potent of them and antagonistic to several clinically pathogenic Gram-positive, Gram-negative bacteria and the fungus *Aspergillus fumigatus* was chosen for further investigation. Analysis of the nucleotide sequence of the 16S rDNA gene of *Bacillus* sp. SAB1 showed a strong similarity (100%) with the 16S rDNA gene of *Bacillus licheniformis* HNL09. The bioactive compounds produced by *Bacillus licheniformis* SAB1 (GenBank accession number: DQ071568) were identified as indole (**1**), 3-phenylpropionic acid (**2**) and a dimer 4,4′-oxybis[3-phenylpropionic acid] (**3**) on the basis of their Fourier Transform Infrared (FTIR), Nuclear Magnetic Resonance (NMR) and Electrospray Ionization Mass Spectrometer (ESI-MS) data. There is a single reference on the natural occurrence of compound **3** from the leaves of a terrestrial herb *Aptenia cordifolia* in the literature, so to the best of our knowledge, this is a first report of its natural occurrence from a marine source. The recovery of bacterial strains with antimicrobial activity suggests that marine-invertebrates remain a rich source for the isolation of culturable isolates capable of producing novel bioactive secondary metabolites.

## 1. Introduction

Sponges are the most primitive of multicelled animals and have existed for more than 800 million years [1]. They are known to produce secondary metabolites which play a decisive ecological role, protecting them against potential invaders, predators or other competitors [2]. Bacteria growing on the surface of sponges live in a highly competitive environment in which access to space and nutrients are limited [3,4]. Secondary metabolites produced by sponge-associated bacteria far exceed those produced by planktonic bacteria [5–9]. Several potentially therapeutic compounds identified in sponges have striking similarities to metabolites derived from their associated microorganisms [10–12]. Hence, sponge-associated bacteria become a highly potential source for the production of antibiotic compounds.

The appearence of bacterial resistance to a number of antimicrobial agents is becoming a major health problem worldwide. Secondly, increasing use and misuse, of existing antibiotics in human, veterinary medicine and in agriculture has further aggravated the problem [13]. Common among them are methicillin-resistant *Staphylococcus aureus* (MRSA), penicillin-resistant *Streptococcus pneumoniae*, vancomycin-resistant *Enterococcus* and *Mycobacterium tuberculosis.* It is further stated that, about 70 percent of the bacteria that cause infections in hospitals are resistant to at least one of the drugs most commonly used for treatment [13]. Hence, the need is now felt more than ever before to find new classes of antimicrobials to combat multi drug resistant (MDR) strains especially targeting marine sources for the purpose.

The aim of the present investigation was to isolate culturable marine bacteria associated with sponge *Halichondria* sp. and to determine which of these isolated bacteria produced potentially useful antimicrobial substances. Their activity was tested against clinical pathogens and multidrug resistant bacterial strains. The most potent culture was identified as *Bacillus licheniformis* SAB1, on the basis of nucleotide sequence analysis of 16S rDNA gene. Mass culture of *Bacillus licheniformis* SAB1 for chemical characterization led to the identification of three compounds of which two are known and the third one, which though known as metabolite of a terrestrial herb, *Aptenia cordifolia,* is being reported here for the first time from a marine bacterium.

## 2. Results and Discussion

It has been estimated that over 99% of the marine sponge-associated microbes have yet to be cultured in the laboratory with bacteria isolated from the sponges containing diverse *Bacillus* species being one of the most divergent forms [14].

Crude extracts of marine bacteria isolated from the surface of sponge *Halichondria* sp. were subjected to antimicrobial (antibacterial and antifungal) screening against clinical pathogens. The list of pathogens used for primary screening is shown in Table 1. Bacterial cultures (SAB1, SAB6, and SAB14) showed activity. However, as SAB1 was the most active one, it was taken for further study.

Strain SAB1 formed circular, opaque, raised, smooth colonies on Zobell Marine Agar (ZMA), with entire margin, creamy white in color and colony forming units measuring 3–4mm in diameter. Based on the nucleotide homology and phylogenetic analysis, the marine bacterium (SAB1) was identified to be *Bacillus licheniformis* (showed 100% similarity with *Bacillus licheniformis* HNL09, GenBank Accession No. EU373344).

Mass culture of *B. licheniformis* SAB1 for chemical characterization resulted in a fermentation medium possessing a very strong unpleasant odor and bioassay guided isolation of the crude methanol extract yielded three compounds. Table 2 shows the spectral details of these three compounds.

Compound **1**, with an intense fecal odor, has a molecular formula C_8_H_7_N as deduced from its pseudomolecular ion [M+H]^+^ at m/z 118.054 in the electospray ionization mass spectrum (ESI-MS). The ^1^H-NMR spectrum (Table 2) exhibited two doublets one centered at δ 7.64 (*J* = 7.5 Hz) and the second at δ 7.28 (*J* = 7.8 Hz) and a singlet at δ6.55. A broad signal at δ 8.13 was assigned to –NH proton. The ^13^C NMR spectrum showed the presence of intense six doublets in the region from δ 102.42 to δ 124.13 ppm and two quaternary carbons at δ 127.76 ppm and δ 135.69 ppm. The compound was confirmed to be indole by comparing the NMR spectrum with spectrum 3,121A, Vol III, of the Aldrich Library of ^13^C and ^1^H FT-NMR spectra (Edition 1). The IR spectrum indicated the presence of an -NH group at 3,402 cm^−1^.


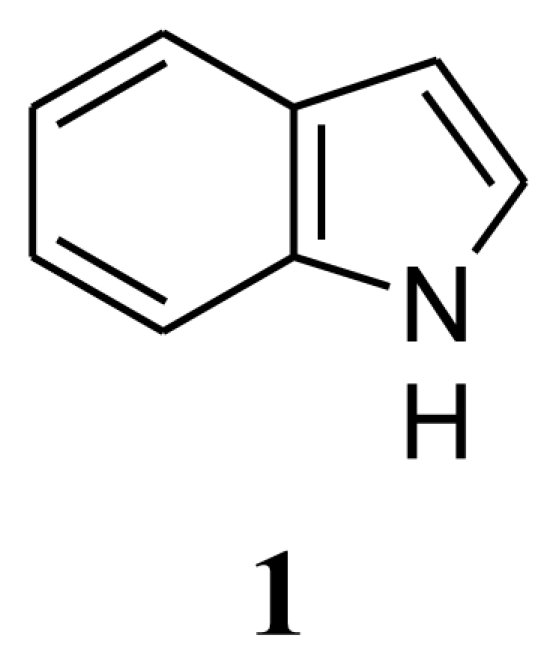


Compound (**2**), a major metabolite from the crude extract of *B. licheniformis* SAB1 was identified as 3-phenylpropionic acid. The electrospray ionization mass spectrum (ESI-MS) showed the presence of a signal at m/z 189 due to [M+K]^+^ and m/z 339 due to [2M+K]^+^ indicating its molecular formula to be C_9_H_10_O_2_. The ^1^H- and ^13^C-NMR data (Table 2) showed signals in the region from δ 7.19 to δ 7.38 for aromatic protons with the corresponding carbons as doublets at δ 126.38, 128.24 and 128.54. The singlet at δ140.13 was attributed to the tetra substituted aromatic carbon and the signal at δ 179.45 was assigned to the carboxyl carbon. The signals due to methylenes α and β to carboxyl group were evident as two triplets in the ^1^H-NMR spectra centered at δ 2.68 and δ 2.96 and the corresponding carbon signal appeared at δ 30.54 and δ 35.61, respectively. The carboxylic acid carbonyl was evident in its infrared spectrum at 1,712 cm^−1^, whereas the presence of signals between 1,604 cm^−1^ and 1,496 cm^−1^ was suggestive of aromaticity that was further confirmed by the presence of corresponding signal in its nuclear magnetic resonance (NMR) spectra. On the basis of the above spectral data the compound has been identified as 3-phenylpropionic acid which was also in good agreement with the spectral data given in Aldrich catalogue of NMR data.


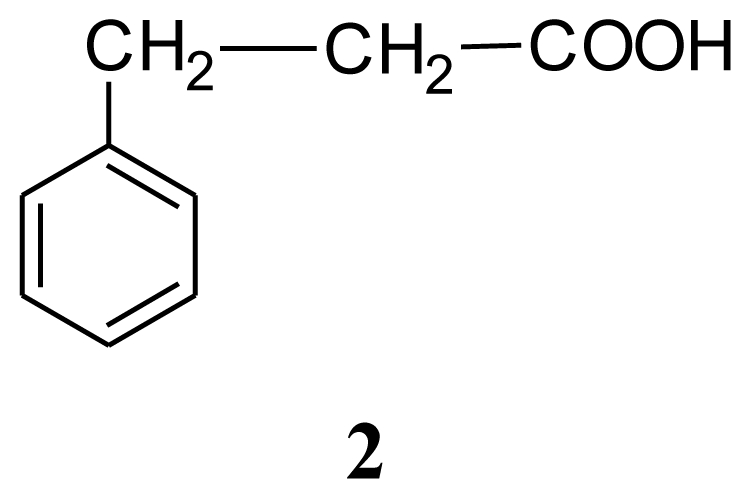


Compound **3**, the most polar of the three showed a pseudomolecular ion peak at m/z 362.1 [M+2Na+2H]^+^ in the ESI-MS spectrum suggesting a molecular formula of C_18_H_18_O_5_. The ^1^H- and ^13^C-NMR data indicated a highly symmetric molecule with its ^1^H-NMR data closely resembling that of compound **2**, but the pattern of substitution in the aromatic ring, *i.e.*, two doublets at δ 6.75 (d, *J* = 8.4 Hz) and δ 7.07 (d, *J* = 8.4Hz) was suggestive of *para* substitution in the ring. Eight aromatic protons were present as two *ortho*-coupled protons and eight methylene protons as two triplets in aliphatic region. The ^13^C-NMR spectrum (Table 2) showed only seven carbon signals. The signal at δ 154.11 indicated that the one substituent in the ring could be a hydroxyl group. The DEPT spectrum showed two methylenes, and two methines. The intense signal at m/z 362.0607 [M+2Na+2H]^+^, suggested that the molecule must be a symmetric dimer with an ether bridge in between. From this spectral data compound 3 was assigned to be 4,4′-oxybis[3-phenylpropionic acid].


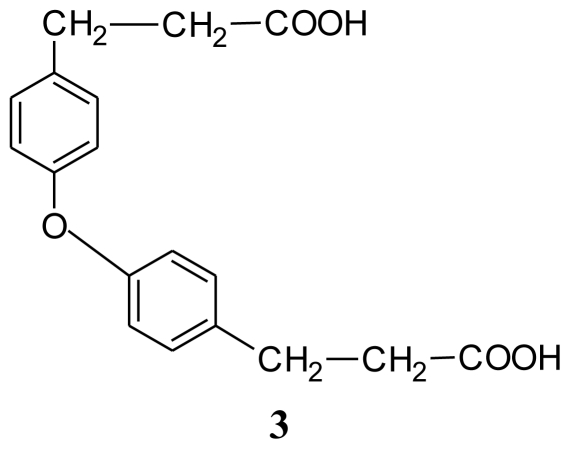


The spectral data of compound **3** agree well with that of apteniol A, an oxyneolignan from the leaves of *Aptenia cordifolia*, a terrestrial perennial herb belonging to the family Aizoaceae, native to South Africa but now largely diffused throughout Europe [15]. There is no literature available to show that it was ever reported earlier from any marine source. Hence, the present study becomes the first report of this compound from a marine bacterium *Bacillus licheniformis* SAB1, and it is also a first report of its antimicrobial activity, as it showed significant antifungal activity against *Aspergillus fumigatus* (7–10 mm zone) and moderate antibacterial activity against *Vibrio cholerae* and *Salmonella typhi*. Apteniol A was tested for its phytotoxicity on the seeds of *Lactuca sativa* at concentrations ranging between 10^−4^ and 10^−7^ M and it was found that apteniol A reduced root elongation by 40% when compared to the control.

Antimicrobial activity of the crude extract, pure compounds, positive (standard antibiotics) and negative (solvent) controls are summarized in Table 3. The crude methanol extract of strain SAB1 showed significant antibacterial activity against *Pseudomonas aeruginosa*, with an inhibition zone between 7–10 mm, moderate (4–6 mm) antibacterial activity against *Staphylococcus aureus*, and antifungal activity against *Aspergillus fumigatus*. Mild inhibitory activity was exhibited by all the test strains under study, except *E coli*, *Shigella flexineri*, *Klebsiella* sp., multi drug resistant (MDR) *Salmonella typhi* and fungal pathogen *Cryptococcus neoformans*. Compound **1** showed significant antibiotic activity against bacterial pathogens: methicillin sensitive *Staphylococcus aureus*, *Salmonella typhi*, and fungal pathogen: *Candida albicans* (inhibition zone 7–10 mm each). Moderate activity was exhibited against *Pseudomonas aeruginosa*, methicillin resistant *Staphylococcus aureus* and fungus *Rhodotorula* sp. (4–6 mm). Weak activity was observed against MDR *Streptococcus pyogenes,* MDR *Acinetobacter* sp. and *Aspergillus niger* (1–3 mm) respectively. Clinical pathogens *Escherichia coli*, *Shigella flexineri*, *Klebsiella* sp., *Vibrio cholerae*, *Salmonella typhi* and the fungal pathogen *Aspergillus fumigatus* and *Cryptococcus neoformans* were insensitive to indole. Compound **2** (3-phenylpropionic acid) showed significant antifungal activity against *Rhodotorula* sp. (7–10 mm inhibition zone) and moderate antifungal activity against *Candida albicans* and *Aspergillus niger* (4–6 mm). On the other hand, only moderate antibacterial activity was shown against *Pseudomonas aeruginosa*, drug sensitive *Staphylococcus aureus*, *Salmonella typhi*, MDR-*Streptococcus pyogenes*, MDR-*Acinetobacter* sp. and methicillin resistant *Staphylococcus aureus*. The remaining pathogens were insensitive to compound **2**. Similar report on antifungal properties of this compound was reported by Mao *et al.* [16], who describe its antifungal activity, inhibiting growth of *D. bryoniae*, *B. cinerea*, *Pestalotiopsis* sp. and *C. gloeosporioides*. Narayana and co-workers [17] reported on its antimicrobial activity against different test microorganisms including bacteria (*Pseudomonas aeruginosa*, *P. fluorescens*, *B. subtilis*, *Escherichia coli* & *Proteus vulgaris*) and fungi (*F. udum*, *Aspergillus flavus*, *Penicillium citrinum*, *Candida albicans* & *A. niger*). Compound **3**, 4,4′-oxybis[3-phenylpropionic acid], showed significant antifungal activity against *Aspergillus fumigatus* (7–10 mm zone) and moderate antibacterial activity against *Vibrio cholerae* and *Salmonella typhi* (Table 3).

Although, the antibiotic activity of marine bacteria is well-known and has been demonstrated in a number of studies [18–21], the vast diversity of microorganisms in the marine niches [22], continue to yield many novel bioactive compounds. Hence, exploration of biotechnological potentials of microbes associated with invertebrates still remains a very important and untapped resource. Modification of culture media and new cultivation methods are clearly needed for better exploration of the biotechnological potential of invertebrate-associated microbes.

## 3. Experimental Section

### 3.1. Isolation of Bacteria Associated with Marine Sponge

All the bacterial strains used in this study were obtained from an orange-colored sponge, *Halichondria* sp. collected off Gujarat, West Coast of India, from a depth of 10 m by SCUBA diving. The sponge sample soon after collection was transferred to a sterile polyethylene bag and transported under frozen condition to the laboratory for the isolation of associated microbes. On reaching the laboratory, the invertebrate was thawed and cut aseptically into small pieces (2 × 2 cm) using a sterile scalpel. The pieces were freed from adhering particles by vortexing twice for 20 seconds with 2 mL of sterile seawater. The seawater was decanted and replaced with methanol, which was once again replaced with sterile seawater with continued vortexing between washings. Finally, samples in sterile seawater were homogenized using sterilized mortar and pestle in a Laminar flow hood. The homogenate was serially diluted up to 10^−3^ dilutions and then spread plated on Zobell Marine Agar (ZMA) plates which contained ketoconazole (100 μg/mL) as an antifungal agent. The plates were incubated at room temperature (28 ± 2 °C) for 2 days till visual growth of culture was observed. Single bacterial colonies were isolated on the basis of distinct colony morphologies from the Zobell Marine Agar (ZMA) plates. Colonies were selected on the basis of uniqueness relative to other plates and ease to select single colonies. Isolates were maintained on ZMA agar slants at 4 °C until use.

### 3.2. Polymerase Chain Reaction

Genomic DNA was isolated from the pure SAB1 culture pellet. Using consensus primers, the ~1.5 kb 16S rDNA fragment was amplified using Taq DNA polymerase. PCR product was bi-directionally sequenced using primer 27f (5′-AGAGTTTGATCCTGGCTCAG-3′), paired with 1492r (5′-TACGGCTACCTTGTTACGACTT-3′) and an internal primer. Sequence data of the strain SAB1 generated in this reaction was aligned with the 16S rDNA sequence of other closely related *Bacillus* species retrieved from the Gene Bank database and analyzed for finding the closest homologues for the microbes. After sequence alignment it was subjected to blast in NCBI database and then preceded with the alignment of the sequences in RDP database (GeNei; NCBI GenBank and RDP database). The alignment demonstrated that the strain was *Bacillus licheniformis* (GenBank accession number DQ071568) as it showed 100% similarity to *Bacillus licheniformis* HNL09 (GenBank accession number EU373344).

### 3.3. Cultivation of Bacterial Isolates for Screening

The isolated bacteria were subcultured on Zobell Marine Agar (ZMA) plates and incubated at 28 ± 2 °C for two days. A loopful of the bacterial culture from the plate was inoculated into 100 mL of Zobell Marine Broth (ZMB) prepared in sterile seawater (in duplicate) and incubated on a shaker at 28 ± 2 °C for 48 h. At the end of incubation period, the fermentation medium was individually centrifuged at 7000× g for 20 minutes to separate cell mass from the fermentation medium. The resulting medium, free from cells, was concentrated under vacuum and the methanol extract of the same was used for primary screening.

### 3.4. Agar Disc Diffusion Assay

Antibiotic screening of the crude extracts as well as the isolated pure compounds was performed by the disc diffusion assay as described earlier [23], against 16 clinical pathogens (listed in Table 1). The bacterial pathogens are numbered from B1 to B7, multi drug resistant (MDR) bacteria from D1 to D4 and fungal pathogens from F1 to F5. Briefly, sterilized (121 °C for 15 min) Whatman filter paper discs measuring 6 mm diameter were loaded with the sample of known concentration (100 μg/disc for crude extracts, 50 μg/disc for pure compounds). These discs impregnated with the test samples were placed on Mueller Hinton Agar plates seeded with test organisms. Positive and negative controls using standard antibiotics (ketoconazole–antifungal; and streptomycin antibacterial) and solvent discs were also run simultaneously. After 24 hours of incubation at 37 °C, the diameter of each zone of growth inhibition was measured in millimeters to obtain a semi quantitative determination of the antibiotic nature of the extracts. The assay was carried out in duplicates.

### 3.5. Cultivation of Sponge-Associated Bacterium (SAB1) for Secondary Metabolite Production

Selected bacterial strain (SAB1) showing significant activity during primary screening was mass cultured to obtain sufficient material for chemical characterization. During mass culture, 8 litres (4 × 2 L) of Zobell Marine Broth (ZMB) comprising of peptone (5 g/L), yeast extract (1 g/L), KH_2_PO_4_ (0.1 g/L) dissolved in seawater was used. After introducing the inoculum of strain SAB1, the flasks were incubated for 72 h at room temperature (27 ± 2 °C) on a shaker. At the end of the incubation period, the cells were separated from the fermentation medium by centrifuging at 7000× g for 20 minutes. The cell-free supernatant medium was concentrated to dryness in a lyophilizer at −80 °C.

### 3.6. Extraction and Purification of Active Metabolites

The crude methanolic extract of the lyophilized culture filtrate (190 mg) was fractionated on a silica gel (60–120 mesh) column with increasing concentrations of ethyl acetate (EA) in petroleum ether (PE) as eluant. Eluates collected in 10 mL fractions were pooled together based on their TLC profile using ethyl acetate-petroleum ether (15:85, v/v) as developing solvent system for compounds **1** and **2** and ethyl acetate-petroleum ether (30:70, v/v) for compound **3**. Spots were visualized by spraying with 5% sulphuric acid in methanol followed by heating of the TLC plate in an oven for 2 minutes at 110 °C. The compounds were eluted in the order of their polarity with compound **1** (49 mg, *R*_f_ = 0.36) being eluted first followed by compound **2** (60 mg, *R*_f_ = 0.2) and the most polar compound **3** (13 mg, *R*_f_ = 0.12) being eluted last.

### 3.7. Analytical Methods/Instrumentation

Lyophilization of sample was carried out in a Christ, ALPHA 2–4 LD plus instrument, NMR spectra were recorded at 23 °C on a Bruker Avance AC-300 spectrometer, operating at 300 and 75 MHz for ^1^H and ^13^C, respectively, using tetramethylsilane (TMS) as an internal standard. Mass data was recorded on an Electrospray Ionization Tandem Mass Spectrometer (ESI/MS-MS) using a QSTAR XL System mass spectrometer from Applied Biosystems. Infra-red spectra were recorded on a FTIR-Shimadzu instrument. TLC was performed on aluminium sheets pre-coated with silica gel 60 F_254_ (Merk KgaA, Damstadt, Germany, Cat No. 1.05554). All the solvents used were glass distilled.

## 4. Conclusions

The marine bacterium, *Bacillus licheniformis* SAB1, isolated from a *Halichondria* sp. sponge and identified by its 16S rDNA is a circular Gram-positive bacterium. It is a motile, spore forming facultative anaerobe belonging to *B. subtilis* group of the Bacilli. Primary screening of the cell free culture filtrate showed significant antimicrobial activity. Mass culture of *B. licheniformis* SAB1, for chemical characterization led to the isolation and identification of three compounds using ^1^H- and ^13^C Nuclear Magnetic Resonance (NMR), Electrospray Ionization Mass Spectrometer (ESI-MS) and Fourier Transform Infrared (FTIR) spectrometers as indole (**1**), 3-phenylpropionic acid (**2**) and 4,4′-oxybis[3-phenylpropionic acid] (**3**). Antibacterial and antifungal activities of these compounds are also reported. The isolation of compound **3**, 4,4′-oxybis[3-phenylpropionic acid], from a marine source, as well as its antimicrobial activity is reported here for the first time.

## Figures and Tables

**Table 1:** List of clinical pathogens used for antibiotic screening.

Sr. No.	Isolate no.	Nature of pathogen	Name of pathogen	Disease it causes
1.	B1	Bacterial pathogens	*Escherichia coli*	Gastrointestinal infection
2.	B2		*Pseudomonas aeruginosa*	Urinary tract infection
3.	B3		*Staphylococcus aureus*	Skin infection
4.	B4		*Salmonella typhi*	Typhoid
5.	B5		*Shigella flexineri*	Gastrointestinal infection
6.	B6		*Klebsiella* sp.	Urinary tract infection
7.	B7		*Vibrio cholerae*	Cholera
8.	F1	Fungal pathogens	*Aspergillus fumigatus*	Skin infection
9.	F2		*Rhodotorula* sp.	Skin infection
10.	F3		*Candida albicans*	Candidiasis
11.	F4		*Cryptococcus neoformans*	Skin infection
12.	F5		*Aspergillus niger*	Skin infection
13.	D1	Multi-drug resistant bacteria	*Streptococcus pyogenes*	Skin infection
14.	D2		*Acinetobacter* sp.	Urinary tract infection
15.	D3		*Salmonella typhi*	Typhoid
16.	D4		Methicillin Resistant *Staphylococcus aureus*	Skin infection

**Table 2:** ^1^H- and ^13^C-NMR data of Compounds **1**, **2** and **3**.

Compound 1: indole			Compound 2: 3-phenylpropionic acid			Compound 3: 4,4′-oxybis(3-phenylpropionic acid)		
Position	^1^HNMR	^13^CNMR	Position	^1^HNMR	^13^CNMR	Position	^1^HNMR	^13^CNMR
1	8. 13 (b,s)	-	1	-	179.45	1,1′	-	132.46
2	7. 64 (d, 7.5Hz)	124.13	2	2.96(t,7.5,7.8Hz)	35.61	2,6,2′,6′	7.07(d, 8.4Hz)	115.43
3	7. 28 (d, 7.8Hz)	121.89	3	2.68(t, 7.5,7.8Hz)	30.54	3,5,3′,5′	6.75(d, 8.4Hz)	129.48
4	6. 55 (s)	102.42	1′	-	140.13	4,4′	-	154.11
5	7.09–7.23 (3H, m)	120.65	2′,6′	7.19–7.38(5H,m)	128.54	7,7′	2.89(t, 7.5,7.8Hz)	29.88
6		119.74	3′,5′		128.24	8,8′	2.64(t, 7.5,7.8Hz)	34.49
7		111.01	4′		126.38			
8		135.69						
9		127.76						

**Table 3:** Antibiotic activity of crude, and pure compounds (Comp **1**- indole; Comp **2** – 3-phenylpropionic acid; Comp **3**- 4,4′-oxybis(3-phenylpropionic acid) isolated from *Bacillus licheniformis* SAB1.

No.	Pathogens used for study	Crude Extract (100μg/disc)	Comp 1 (50μg/disc)	Comp 2 (50μg/disc)	Comp 3 (50μg/disc)	STD Antibiotics (50μg/disc)	Solvent Methanol
B1	*Escherichia coli*	-	-	-	-	++++	-
B2	*Pseudomonas aeruginosa*	+++	++	++	-	+++++	-
B3	*Staphylococcus aureus*	++	+++	++	-	+++	-
B4	*Salmonella typhi*	+	+++	++	++	++	-
B5	*Shigella flexineri*	-	-	-	-	+++++	-
B6	*Klebsiella* sp.	-	-	-	-	+++++	-
B7	*Vibrio cholerae*	+	-	-	++	+++++	-
D1	*Streptococcus pyogenes*	+	+	++	-	++	-
D2	*Acinetobacter* sp.	+	+	++	-	-	-
D3	*Salmonella typhi*	-	-	-	-	-	-
D4	Methicillin Resistant *S.aureus*	+	++	++	-	++	-
F1	*Aspergillus fumigatus*	++	-	-	+++	++++	-
F2	*Rhodotorula* sp.	+	++	+++	-	-	-
F3	*Candida albicans*	+	+++	++	-	-	-
F4	*Aspergillus niger*	+	+	++	-	-	-
F5	*Cryptococcus neoformans*	-	-	-	-	-	-

- no zone of growth inhibition;

+ 1–3mm zone of inhibition;

++ 4–6mm zone of inhibition;

+++ 7–10mm zone of inhibition;

++++ 11–15mm zone of inhibition;

+++++ 16–22mm zone of inhibition; Antibiotics: Streptomycin/Ketoconazole.
